# Navigating the U.S. regulatory landscape for neurologic digital health technologies

**DOI:** 10.1038/s41746-024-01098-5

**Published:** 2024-04-12

**Authors:** Neil A. Busis, Dilshad Marolia, Robert Montgomery, Laura J. Balcer, Steven L. Galetta, Scott N. Grossman

**Affiliations:** 1 Department of Neurology, NYU Grossman School of Medicine, New York, NY USA; 2 NYU Langone Health System, New York, NY USA; 3 Clinical Affairs and Ambulatory Care, NYU Langone Health System, New York, NY USA; 4 Department of Population Health, NYU Grossman School of Medicine, New York, NY USA; 5 Department of Ophthalmology, NYU Grossman School of Medicine, New York, NY USA

**Keywords:** Health policy, Diagnostic markers

## Abstract

Digital health technologies (DHTs) can transform neurological assessments, improving quality and continuity of care. In the United States, the Food & Drug Administration (FDA) oversees the safety and efficacy of these technologies, employing a detailed regulatory process that classifies devices based on risk and requires rigorous review and post-market surveillance. Following FDA approval, DHTs enter the Current Procedural Terminology, Relative Value Scale Update Committee, and Centers for Medicare & Medicaid Services coding and valuation processes leading to coverage and payment decisions. DHT adoption is challenged by rapid technologic advancements, an inconsistent evidence base, marketing discrepancies, ambiguous coding guidance, and variable health insurance coverage. Regulators, policymakers, and payers will need to develop better methods to evaluate these promising technologies and guide their deployment. This includes striking a balance between patient safety and clinical effectiveness versus promotion of innovation, especially as DHTs increasingly incorporate artificial intelligence. Data validity, cybersecurity, risk management, societal, and ethical responsibilities should be addressed. Regulatory advances can support adoption of these promising tools by ensuring DHTs are safe, effective, accessible, and equitable.

## Introduction

A digital health technology (DHT) is a “*system that uses computing platforms, connectivity, software, and/or sensors, for healthcare and related uses*^1^.” To support the appropriate adoption of DHTs, regulators must verify that they meet their potential to improve healthcare. This article examines the processes for navigating the regulatory framework in the United States (U.S.) for neurological DHTs, including marketing authorization, determination of clinical effectiveness, implementation, coding, coverage, and reimbursement.

DHTs can assess aspects of neurologic health, including motor function, sleep, cognition, speech, electroencephalography (EEG), pupils, eye movements, and other domains, alone or in combination^2–8^. For example, rapid picture naming^9^ evaluates a variety of functions simultaneously, including attention, language, cognition, and eye movements. DHTs can quantify results from the traditional neurologic examination and detect findings unobtainable by other methods. Certain DHTs allow healthcare providers to carry out specific examination procedures that would typically require a subspecialist. DHTs can send data they collect to both healthcare professionals and AI systems to assist in making medical decisions^10^. DHTs span a wide spectrum^11^ from unregulated direct-to-consumer “lifestyle” products to complex medical devices that could pose a risk of illness or injury and are subject to regulatory oversight along with coding and billing guidelines.

## The role of the FDA

The U.S. Food & Drug Administration (FDA) regulates medical devices to ensure that they are safe for the patient and effective for the stated treatment^12^. FDA clearance or approval, or having a path to receive it, is essential to implement a viable DHT program. The FDA Medical Devices Advisory Committee^13^ consists of 18 panels, including the Neurological Devices Panel, that advise the Commissioner about issues related to the safety and effectiveness of medical devices.

The FDA follows a five-step process from device discovery and concept through review and post-market safety monitoring. Devices are initially classified by the degree of risk imposed on the consumer. Then, applicants must build preclinical prototypes for investigation in non-human laboratory environments. The pathway to clearance requires classification centered on the degree of risk. The FDA may convene an advisory committee of independent experts at a public meeting if it lacks internal expertise in a particular content area.

FDA medical device review is iterative. While premarket clinical trials provide data on a medical device’s safety and effectiveness, new concerns may emerge once the device is on the market^14^.

Whether a specific DHT has been cleared or approved by the FDA as medical device can be determined by searching the FDA’s publicly available Medical Device databases^15^. If the DHT is not approved or cleared as a medical device, then the process of seeking such approval or clearance begins with confirming that the DHT does indeed meet the legal definition of a medical device.

The next step is ensuring that an appropriate product classification exists for the DHT by checking the FDA Product Classification databases^16^. Examples of product classifications include stents, blood pressure cuffs, and automatic event detection software for EEG. As illustrated by these examples, a medical device is not restricted to hardware but also includes software, referred to by the FDA as either “Software as a Medical Device (SaMD)” or “Software in a Medical Device (SiMD”).

Criteria outlined in the Food, Drug & Cosmetic Act determine whether to regulate software as a medical device. A software function is classified as a medical device unless it meets these four criteria^17^:

1. It is not intended to acquire, process, or analyze a medical image or a signal from an in vitro diagnostic device or a pattern or signal from a signal acquisition system;
2. It is intended for the purpose of displaying, analyzing, or printing medical information about a patient or other medical information, such as peer-reviewed clinical studies and clinical practice guidelines;
3. It is intended for the purpose of supporting or providing recommendations to a healthcare professional (“HCP”) about prevention, diagnosis, or treatment of a disease or condition; and
4. It is intended for enabling such HCP to review the basis for the recommendations independently that such software presents so that it is not the intent that the HCP rely primarily on any of such recommendations to make a clinical diagnosis or treatment decision regarding an individual patient.

The FDA’s decision to regulate stems from whether the software provides data that is analytical, specific to a particular patient, and determinative in generating an individual treatment plan.

Once a DHT has been determined to be a medical device, but one that has not been approved or cleared by the FDA, the subsequent regulatory pathway is determined by whether the DHT would be classified as a Class I (e.g., tongue depressor), Class II (e.g., blood pressure cuffs), or Class III (e.g., pacemakers) device. The FDA bases its tiered classification structure on the level of risk, invasiveness, and potential impact on patient health. The FDA gives “clearances” for United States Class I and Class II medical devices and “approvals” for Class III medical devices.

The greater the safety risk a device poses to a patient, the more rigorous the review. The highest-risk devices go through a Premarket Approval (PMA). Devices that are low to moderate risk and for which there is an existing device that is legally marketed (called a “predicate” device) will go through a Premarket Notification (510(k))^18^. A device that is low to moderate risk and for which there is no predicate will need a De Novo Classification Request. For most medical devices, securing clearance from the FDA simply requires showing that they are “substantially equivalent” to a predicate device. If a DHT is considered a Class II Medical Device, the level of rigor involved in the clearance is not equivalent to that for a Class III medical device, and neither the provider nor the patient should assume extensive review. The clearance is more a statement of “no harm” and is not an official endorsement. Novel DHTs have largely been “cleared” by the FDA to date, indicating the FDA’s willingness to consider these DHTs as potentially low-risk. Conversely, the FDA has issued safety warnings when devices that are not FDA-approved purport to provide important health information^19^. The FDA collaborates with international partners on digital health initiatives^20^, and other countries formulate their own policies^21^.

## The CPT® – RUC – CMS Cycle

DHTs evolve from idea to reimbursable entity in a multi-step process starting with FDA approval^22,23^. Current Procedural Terminology (CPT®) codes are the uniform language for coding medical services and procedures^24^. Category I codes describe widely used and accepted technologies, services, and procedures. They are included in the Medicare Physician Fee Schedule (MPFS)^25^. Category III codes are a temporary set of codes for emerging technologies, services, and procedures. If covered, they may be reimbursed on a case-by-case basis. The entire code set is updated yearly. Societies, individuals, and industry recommend new or revised CPT® codes to the CPT® Editorial Panel.

The Relative Value Scale Update Committee (RUC)^26^ assigns relative value units (RVUs) to each new or revised Category I CPT® code. These RVUs quantify physician work, practice expense, and liability insurance necessary to perform the service or procedure described by the code. The RUC sends its determinations to the Centers for Medicare & Medicaid Services (CMS) for consideration of coverage and payment in the next MPFS. Key criteria include whether the service or procedure is medically reasonable and necessary. Other payers use the MPFS to develop their policies.

## Implementing digital health technologies

Key questions to answer when considering a DHT program are whether it will improve the value of care^27^ by increasing quality, enhancing patient and provider experience, or decreasing cost and whether the financial model is sustainable (Box 1). To ensure the accuracy of the information, consult multiple sources before choosing a DHT. There are discrepancies in how certain devices are marketed compared to their submitted 501(k) applications^28,29^. Neurologic DHTs may be reported using several CPT® code families (Box 2). However, mapping a DHT onto a specific CPT® code is not always straightforward, and coding and documentation guidelines can be complex and ambiguous. Currently, DHTs have limited utilization. Payer policies are not aligned, lack transparency, and have inconsistent DHT coverage with varying processes and timelines for incorporating new DHT CPT® codes^30^.

To ensure the successful implementation of the program, it is essential to assemble an interdisciplinary team. Operational teams can resolve device and implementation challenges, establish efficient workflows, and develop information technology needed for the integration and accessibility of data streams and electronic health information. Billing compliance and revenue cycle teams can ensure correct documentation, proper application of CPT® codes, and appropriate reimbursement. Inviting patient and provider feedback can gauge experience and satisfaction using the DHT, device compliance, ease of use, and other measures of program success.

Box 1 Neurologic DHT implementation checklist

**Table:** 

1. Identify patient population
2. Assess the clinical need
3. Develop the financial model
4. Select FDA-approved device
5. Form and consult with cross-functional teams
6. Implement efficient workflows
7. Ensure documentation and billing compliance
8. Invite patient and provider feedback

Box 1 Legend: Topics to consider before, during, and after implementation of a DHT.

Box 2 CPT® Codes pertinent to neurologic DHTs

**Table:** 

**Category I Codes**
Central nervous system evaluation code family (includes cognitive, mental status, and speech testing)
Motion analysis code family
Quantitative pupillometry
Remote physiologic monitoring code family
Remote therapeutic monitoring code family
Sleep medicine testing code family
Electroencephalography code family
Unlisted neurological or neuromuscular diagnostic procedure
**Category III Codes**
Eye-movement analysis
Quantitative sensory testing code family
Automated visual acuity screening
Assessing range of motion, posture, gait, and muscle function using sensors

Box 2 Legend: Examples of Category I and Category III CPT® codes and code families pertinent to neurologic DHTs^24^. This list is not intended to be exhaustive. Use cases where a DHT is not reported separately but is utilized to support an evaluation and management or care management service are also not included. The Unlisted Neurological or Neuromuscular Diagnostic Procedure code can be used for DHTs without more specific Category I CPT® codes, e.g., tremor measurement. Category I CPT® codes are updated annually. Category III CPT® codes are updated semiannually. Up-to-date code numbers and definitions are included in the CPT® manual for the current calendar year.

### Case Study 1: Direct to consumer app suite

A company (https://becarelink.com/) offers a suite of mobile apps for neurological assessments. Consumers are asked to subscribe to apps which can send data to their healthcare professionals. “People are empowered to direct their own healthcare from home through these remote assessments.” These apps:

- “measure your memory, motor skills, walking ability, and more,”
- “enhance your multiple sclerosis (MS) monitoring,”
- “assess potential neurological and neurobehavioral damage caused by water contamination,” and
- provide healthcare professionals with “quantitative data with trending graphics and increase revenues by having additional CPT codes to bill for each encounter.”

The company states it has received FDA Class I clearance for its MS app. Class I clearance is granted to items such as tongue depressors and bandages and, as a rule, does not require premarket notification from the FDA. This case study illustrates direct-to-consumer marketing of a collection of neurologic DHTs for home use largely outside FDA regulatory pathways.

### Case study 2: Eye-movement assessment

An eye-tracking device to aid in the diagnosis of concussion has been approved by the FDA for clinical use and assigned a CPT® code^31^. This DHT underwent testing to demonstrate conformance to FDA standards, including “medical electrical equipment: general requirements for basic safety and essential performance” and “collateral standard: electromagnetic compatibility.” Software and bench performance testing were also performed. The FDA evaluated the device with the primary endpoints of sensitivity and specificity in discriminating the presence or absence of concussion in head-injured patients. The study included positive- and negative-predictive value post-hoc analyses. Pre-specified performance goals of confidence limit greater than 70% were set as the standard for successful discrimination. The pivotal clinical study incorporated into the FDA approval process did not meet these goals. Nevertheless, the FDA deemed that effectiveness analyses demonstrated probable benefit of the device that outweighed the probable risk to the public. The DHT was given a Category III CPT® code^24^ in 2021: “Eye-movement analysis without spatial calibration, with interpretation and report.” This code is scheduled to sunset in January 2026 unless it is extended or converted to a Category I code.

Although technically FDA-approved, measurements generated by such portable eye-tracking devices should be validated^32^ by comparisons with results from laboratory-standard eye trackers and with simple and inexpensive clinical performance measures that can be administered by trained individuals such as sports parents, including rapid automatized naming (RAN) tasks incorporating pictures and numbers^33–35^. RAN tasks have been utilized for nearly a century and only require paper and pencil or a computerized tablet to administer.

This case study highlights some challenges and drawbacks associated with the current DHT regulatory framework. Even if a portable eye-tracking device was created with the best intentions, was FDA-approved, and given a CPT code, the clinical utility of this technology remains speculative. False reassurance from an insensitive test may put patients at risk. Moreover, the granting of a CPT® code for a service or procedure does not guarantee reimbursement by healthcare insurance plans.

## Conclusions

DHTs have great potential to improve neurologic care^2–8^. However, DHT adoption is challenged by rapid technological advancements, an inconsistent evidence base, marketing discrepancies, ambiguous coding guidance, and variable health insurance coverage. Do FDA approvals and CPT® codes for DHTs always reflect their actual clinical benefits?

Key stakeholders, including the FDA^36^, CPT®^23^, RUC, and CMS, need to evaluate these promising technologies and guide their deployment^37^. This includes striking a balance between patient safety and clinical effectiveness versus the promotion of innovation^38^. FDA approval processes may need to be revised, particularly for DHTs that incorporate artificial intelligence (AI)^39^. Do regulators, policymakers, payers, and their staff have the necessary expertise to evaluate these DHTs in depth? Under what circumstances should independent subject matter experts be consulted? Should the composition of the FDA’s Neurological Devices Panel be changed? What are the best methods for ascertaining whether a DHT possesses substantial therapeutic, diagnostic, or monitoring utility, meriting FDA clearance or approval and potentially qualifying for a Category I CPT® code? How to best determine whether a DHT meets Category I CPT® code volume criteria—i.e., is it “performed by many physicians or other qualified healthcare professionals across the United States… with frequency consistent with the intended clinical use^24^?”

There is also the issue of potentially misaligned incentives. Consumers and entrepreneurs may favor certain DHTs for their convenience and possible reimbursement, even if the supporting evidence for their clinical effectiveness is weak. What are the most effective ways to integrate the results from DHTs with our current practice of neurology?

The continuous monitoring and data collection offered by many DHTs are convenient for patients and caregivers, potentially encourage greater patient compliance, and provide large volumes of data on which to base therapeutic decisions. Which clinical contexts justify continuous monitoring^40^? What safeguards will be put in place to ensure that data collected from DHTs is accurate and validated? Is the current 501(k) process, where only substantial equivalence of a predicate device needs to be proven before approval, adequate?

In the transition towards a more sophisticated understanding of the social determinants of health, healthcare stakeholders, including regulators and policymakers, should consider whether a device is accessible and equitable in addition to being safe and effective^41^. Ease of use and reduced obstacles may increase diversity in the patient pool.

Assuming that DHT systems are working perfectly at baseline, how will the FDA assess the impact of cybersecurity threats, both data breaches and resultant DHT malfunctions? It is unclear if the current 501(k) process addresses these questions. How will regulators and policymakers ensure appropriate risk management and liability allocation? If data indicates potentially life-threatening events, what is the duty of the entity collecting the data to intervene in real-time or engage with the patient for remediation as quickly as possible?

Answers to these questions should be nuanced and thoughtful to ensure patient safety and provider confidence. Healthcare and consumer organizations are collaborating to consider the optimal roles of DHTs^42^. The FDA has begun deliberations on how to regulate DHTs by forming steering committees, including the Digital Health Advisory Committee^43^, convening public meetings, and creating demonstration projects.

The use of DHTs in neurology can become more widespread if their value is demonstrated convincingly. Regulatory advances can support appropriate adoption by ensuring DHTs are safe, effective, accessible, and equitable. Key stakeholders have important roles to play in the evaluation, dissemination, and implementation of DHTs (Boxes 3 and 4).

Box 3 Key takeaway points for regulators, policymakers, and payers

- **Develop and adopt improved DHT evaluation methods:** New sophisticated evaluation methods are needed to keep pace with rapid technological advancements and the incorporation of artificial intelligence in DHTs. These methods should balance the need for innovation with the imperative to maintain high standards of patient safety and clinical effectiveness.
- **Refine regulatory framework for DHT safety and efficacy:** Enhancing DHT regulatory processes, focusing on a risk-based classification, comprehensive premarket evaluations, and rigorous post-market oversight should help ensure their safety and effectiveness.
- **Simplify DHT use with clear coding and coverage guidance:** Straightforward guidance and resolving disparities in insurance coverage to facilitate easier decisions regarding coverage and payment will streamline DHT adoption.
- **Address evidence and marketing inconsistencies for DHT credibility:** Concerted efforts to rectify inconsistencies in the evidence base and to correct marketing discrepancies will enhance the credibility, adoption, and practical application of DHTs in clinical settings.
- **Tackle data integrity and ethical issues for DHT trust:** Addressing concerns related to data validity, cybersecurity, and risk management, as well as societal and ethical considerations, is essential to ensure DHTs are trustworthy, accessible, equitable, and ready for widespread use.

Box 4 Key takeaway points for clinicians

- **DHTs enhance neurological assessments:** They allow for the detailed measurement of traditional examination outcomes, uncover findings that might not be visible otherwise, and enable generalists to perform specialized tests.
- **FDA evaluates DHTs:** Evaluations are based on associated risks and include a detailed review process and ongoing surveillance after the product enters the market to guarantee safe use in healthcare settings.
- **Coding, valuation, coverage cycle:** After FDA approval, DHTs undergo coding, valuation, and insurance coverage determinations by CPT, RUC, and CMS, crucial steps for securing reimbursement.
- **Barriers to DHT adoption:** The rapid evolution of technology, combined with an inconsistent evidence base, marketing variations, and unclear coding and insurance rules, create obstacles to DHT adoption.
- **DHT deployment:** Successful implementation involves identifying patient needs, selecting appropriate devices, forming cross-functional teams, designing effective workflows, ensuring compliance, and gathering feedback from patients and providers.
